# Gene Mutation Spectrum of Thalassemia Among Children in Yunnan Province

**DOI:** 10.3389/fped.2020.00159

**Published:** 2020-04-15

**Authors:** Ti-Long Huang, Tian-Yao Zhang, Chun-Yan Song, Yun-Bi Lin, Bao-Hua Sang, Qing-Ling Lei, Yu Lv, Chun-Hui Yang, Na Li, Xin Tian, Yue-Huang Yang, Xian-Wen Zhang

**Affiliations:** ^1^Department of Hematology, Kunming Children's Hospital, Kunming, China; ^2^Department of Endocrinology and Metabolism, The Second Affiliated Hospital of Harbin Medical University, Harbin, China; ^3^Medical Faculty, Kunming University of Science and Technology, Kunming, China

**Keywords:** thalassemia, gene mutation spectrum, children, Yunnan province, China

## Abstract

**Background:** Thalassemia is an autosomal genetic disorder, found throughout the world. It is still not treatable and create socio economic problems. In this study, we investigated the prevalence and spectrum features of thalassemia in Yunnan Province, the southwestern area of China. During 2014–2018, a total of 3,539 suspected thalassemia children were detected with α- and β-thalassemia mutations by gap-Polymerase Chain Reaction (PCR) and reverse dot blot (RDB) analysis in Kunming Children's Hospital.

**Results:** Of these patients, 1,130 were diagnosed thalassemia gene carriers with a carrying rate of 31.92%. Among them, α-thalassemia was 43.63%, β-thalassemia was 53.98%, cases with both α- and β- thalassemia was 2.39%. In α-thalassemia patients, the most common mutations was –^SEA^/αα (52.13%), followed by –α^3.7^/αα (27.79%), hemoglobin H disease (18.46%), and –α^4.2^/αα (1.62%). Fifteen gene mutations and 30 genotypes were identified in β-thalassemia patients, with the five most common mutations CD17 (A>T) (29.51%), CD41–42 (–TTCT) (27.87%), IVS-II-654 (C>T) (14.92%), CD26 (G>A) (6.89%), and CD26/CD27 (2.62%) accounting for 81.81% of the β-globin gene mutations. Furthermore, we founded two rare mutations CD34 (TGG → TAG) and Int in Chinese populations.

**Conclusions:** Our results suggested that the prevalence and gene mutation spectrum of thalassemia display obviously heterogeneity among children in Yunnan Province. The findings provide the valuable information for premarital and pre-pregnancy screening, prenatal diagnostic services, and designing appropriate prevention programs to control thalassemia for future in this area.

## Introduction

Thalassemia is an inherited autosomal recessive disease resulting from mutations in the α- and β-globin gene clusters on chromosome 16 and chromosome 11, respectively. It is characterized by the absence or reduced synthesis of globin chains of hemoglobin and includes two main types, α- and β- thalassemia (1, 2). It is reported thalassemia is one of the top five kinds of major birth defects (3). To date, there is no ideal treatment for patients with thalassemia major, except bone marrow transplantation (4). Thalassemia major has imposed an enormous burden on society and has serious impact on the quality of life of the population (5).

High prevalence of thalassemia was mainly reported in southern China, Southeast Asia, India, the Middle East, Africa and Mediterranean region (6). However, population in different regions has their own spectrums of thalassemia (7). At present, it is reported thalassemia is widely distributed on the southern China, particularly in the provinces of Guangdong (8), Guangxi (9), and Hainan (10). Yunnan which have a high frequency of thalassemia is located in the southwestern of China at the junction of Guangxi, Guizhou, and Sichuan provinces (11). However, only few studies reported the prevalence and distribution pattern of α- and β-thalassemia in Yunnan provinces, especially in children. This study is the first large-scale investigate to analyze the prevalence and mutations features of thalassemia children in Yunnan Province.

## Materials and Methods

### Subjects

A total of 3,539 children who were suspected of thalassemia were recruited and tested (male 1,843, female 1,696, age range from 0 to 10 years) from January 2014 to December 2018 in Kunming Children's Hospital, Yunnan, China. The study has been approved by the Ethic Committee of Kunming Children's Hospital. Consent forms were signed by the parents.

### Hematological Analysis

Peripheral venous blood (5 mL) was collected into an EDTA anticoagulated tube. Peripheral blood counts and red blood cell indices were determined using the automatic blood cell analyzer (Kobe, Japan).

The children with low mean corpuscular volume (MCV) values (<82 fl) or low mean cell hemoglobin (MCH) values (<27 pg) were considered possible carriers of thalassemia (12).

Hemoglobin electrophoresis was used to analyze the percent of hemoglobin Hbs A, A2, F, and any abnormal hemoglobin variant. The children with low HbA2 (<2.5%) values were considered possibly α-thalassemia carriers, with high HbA2 (>3.5%) values were considered possibly β-thalassemia carriers, respectively (13).

### Genetic Analyses

According to the instructions (14). We used QIAamp DSP DNA Blood Mini Kit to extract DNA from blood. Genomic DNAs of subjects were extracted from peripheral blood lymphocytes by extraction kits according to the manufacturer's instructions. Three known α-thalassemia deletion defects (–^SEA^/αα, –α^3.7^/αα, –α^4.2^/αα) were determined by the gap-polymerase chain reaction (Gap-PCR) technique. 17 known β-globin mutations was detected by PCR-reverse dot blot (PCR-RDB) assay. In order to detect unknown and rare β-globin mutations, β-globin gene of all the samples was directly sequenced.

## Results

### The Characteristic of Thalassemia in Yunnan Province

During 2014 to 2018, 1,130 of the 3,539 suspected children were diagnosed thalassemia gene carriers with a carrying rate of 31.92% (1,130/3,539). Among them, α-thalassemia children were 43.63% (493/1,130), β-thalassemia children were 53.98% (610/1,130), children with both α- and β-thalassemia were 2.36% (27/1,130). Three α-globin gene deletion and five genotypes were detected in α-thalassemia children. Fifteen gene mutations and 30 genotypes were identified in β-thalassemia children (Tables 1–3).

**Table 1:** Frequency of α-thalassemia mutations in children in the Yunnan province.

**Mutation**	**Type**	**Case (*n*)**	**Frequency (%)**
Heterozygote		402	81.54
–^SEA^/αα	α^0^/α	257	52.13
–α^3.7^/αα	α^+^/α	137	27.79
–α^4.2^/αα	α^+^/α	8	1.62
Compound heterozygote		91	18.46
–^SEA^/–α^3.7^	α^0^/α^+^	81	16.43
–^SEA^/–α^4.2^	α^0^/α^+^	10	2.03
Total		493	100

*α, normal production of the α-globin polypeptide chain; α^0^, no production of the α-globin polypeptide chain; α^+^, reduced production of the α-globin polypeptide chain*.

**Table 2:** Frequency of β-thalassemia mutations in children in the Yunnan province.

**Mutation**	**Type**	**Case (n)**	**Frequency (%)**
Heterozygote		508	83.29
CD17/β^N^	β^0^/β^N^	180	29.51
CD41–42/β^N^	β^0^/β^N^	170	27.87
IVS-II-654/β^N^	β^0^/β^N^	91	14.92
CD26/β^N^	β^E^/β^N^	42	6.89
CD71–72/β^N^	β^0^/β^N^	15	2.46
IVS-I-I/β^N^	β^0^/β^N^	4	0.66
−28/β^N^	β^+^/β^N^	3	0.49
−29/β^N^	β^+^/β^N^	1	0.16
CD34/β^N^		1	0.16
CD43/β^N^	β^0^/β^N^	1	0.16
Compound heterozygote		82	13.44
CD26/CD27	β^E^/β^0^	16	2.62
CD41–42/CD17	β^0^/β^0^	15	2.46
CD26/IVS-II-654	β^E^/β^+^	11	1.80
CD41–42/CD26	β^0^/β^E^	10	1.64
CD17/−28	β^0^/β^+^	5	0.82
CD27/CD28	β^0^/β^0^	4	0.66
CD17/IVS-II-654	β^0^/β^+^	4	0.66
CD41–42/IVS-II-654	β^0^/β^0^	3	0.49
CD41–42/CD43	β^0^/β^0^	3	0.49
CD41–42/−28	β^0^/β^+^	3	0.49
CD14–15/β^N^	β^0^/β^N^	3	0.49
CD26/−28	β^E^/β^+^	1	0.16
CD26/CD43	β^E^/β^0^	1	0.16
CD71–72/CD26	β^0^/β^E^	1	0.16
CD41–42/IVS-I-I	β^0^/β^0^	1	0.16
INT/β^N^	β^0^/β^N^	1	0.16
Homozygote		20	3.29
CD17/CD17	β^0^/β^0^	13	2.13
CD41–42/CD41–42	β^0^/β^0^	4	0.66
IVS-II-654/IVS-II-654	β^0^/β^0^	2	0.33
CD26/CD26	β^E^/β^E^	1	0.16
Total		610	100

*β^N^, normal production of the β-globin polypeptide chain; β^0^, no production of the β-globin polypeptide chain; β^+^, impaired production of the β-globin polypeptide chain; β^E^, Codon26*.

**Table 3:** Distribution of carriers of both α- and β-thalassemia genotypes in children in Yunnan province.

**Mutation**	**Type**		**Case (n)**	**Frequency (%)**
–^SEA^/αα, CD17/β^N^	α^0^/α	β^0^/β^N^	4	14.81
–^SEA^/–α^3.7^, CD26/β^N^	α^0^/α^+^	β^E^/β^N^	4	14.81
–α^3.7^/αα, CD17/β^N^	α^+^/α	β^0^/β^N^	3	11.11
–^SEA^/αα, CD41–42/β^N^	α^0^/α	β^0^/β^N^	3	11.11
–^SEA^/αα, CD26/β^N^	α^0^/α	β^E^/β^N^	2	7.41
–α^3.7^/αα, CD41–42/β^N^	α^+^/α	β^0^/β^N^	2	7.41
–α^4.2^/αα, CD41–42/CD17	α^+^/α	β^0^/β^0^	1	3.70
–^SEA^/–α^3.7^, CD17/β^N^	α^0^/α^+^	β^0^/β^N^	1	3.70
–^SEA^/αα, CD41–42/CD17	α^0^/α	β^0^/β^0^	1	3.70
–^SEA^/αα, CD43/IVS-II-654	α^0^/α	β^0^/β^0^	1	3.70
–^SEA^/–α^4.2^, CD41–42/β^N^	α^0^/α^+^	β^0^/β^N^	1	3.70
–α^3.7^/αα, CD17/−28	α^+^/α	β^0^/β^+^	1	3.70
–α^3.7^/αα, CD41–42/CD17	α^+^/α	β^0^/β^0^	1	3.70
–α^3.7^/αα, IVS-II-654/β^N^	α^+^/α	β^0^/β^N^	1	3.70
–α^3.7^/αα, CD26/β^N^	α^+^/α	β^E^/β^N^	1	3.70
Total			27	100.00

*α, normal production of the α-globin polypeptide chain; α^0^, no production of the α-globin polypeptide chain; α^+^, reduced production of the α-globin polypeptide chain. β^N^, normal production of the β-globin polypeptide chain; β^0^, no production of the β-globin polypeptide chain; β^+^, impaired production of the β-globin polypeptide chain; β^E^, Codon26*.

### The Prevalence and Mutation Spectrum of α-Thalassemia

13.93% (493/3,539) children were diagnosed as being carriers of α-thalassemia (Table 1), three known mutations in the α-globin gene were found, in which the –^SEA^/αα deletion was the most prevalent genotype, accounting for over one half of the mutations (52.13%), followed by –α^3.7^/αα (27.79%), hemoglobin H disease (18.46%), and –α^4.2^/αα (1.62%). Meanwhile, five α-globin genotypes were detected, including 402 children (81.54%) with heterozygous mutations and 91 children (18.46%) with compound heterozygous mutations. Overall, the first three mutations accounted for 98.38% of all α-globin gene mutations.

### The Prevalence and Mutation Spectrum of β-Thalassemia

17.24% (610/3,539) children were carriers of the β-thalassemia, as shown in Table 2. Genetic analysis revealed 15 kinds of mutations and 30 genotypes, including 508 (83.29%) heterozygotes, 82 (13.44%) compound heterozygotes, and 20 (3.29%) homozygotes. The first five most common mutations are CD17 (A>T) (29.51%), CD41–42 (–TTCT) (27.87%), IVS-II-654 (C>T) (14.92%), CD26 (6.89%), and CD26/CD27 (2.62%) accounting for 81.81% of β-thalassemia genotypes. In addition, we founded two particularly rare mutations CD34 (TGG → TAG) and Int in Chinese populations.

### The Prevalence and Mutation Spectrum of Both α- and β-Thalassemia

The distributions of mutations and hematological features are shown in Table 3. 0.76% (27/3,539) children were both α- and β-thalassemia, 15 genotypes were discovered. The top four genotypes of thalassemia were –^SEA^/αα, CD17/N (14.81%); –^SEA^/–α^3.7^, CD26/N (14.81%); –α^3.7^/αα, CD17/N (11.11%); –^SEA^/αα, CD41–42/N (11.11%).

## Discussion

In the present study, we have investigated the prevalence and mutation characteristics of thalassemia among the children in Yunnan province, the southwestern of China. Among 3,539 suspected children, 1,130 were diagnosed with thalassemia, 493 had α-thalassemia, 610 had β-thalassemia, and 27 were carriers with both α- and β-thalassemia. –^SEA^/αα was the most frequent genotype in α-thalassemia and CD17 was the most frequent genotype in β-thalassemia.

It is reported that about 7% of the world's population are carriers of a globin gene mutation (7). In mainland China the overall prevalence of α-, β-, and α+β-thalassemia was 7.88, 2.21, and 0.48%, respectively (15). Our study showed α-, β-, and α+β-thalassemia was 13.93, 17.24, and 0.76%, respectively (Tables 1–3). Compared with the overall prevalence, the higher thalassemia prevalence in Yunnan province showed in our study. The reason may be concerned to the selection of the subjects who detected thalassemia gene mainly due to microcytic hypochromic anemia, hemolytic anemia of unknown origin, not random crowd, which is also the limitation of this study. In our study, there was more β-thalassemia children than α-thalassemia children which is similar to some studies of the thalassemia in China (16).

Previous studies have shown that α-thalassemia is mainly caused by three types of gene deletions, including the Southeast Asian deletion (–^SEA^/αα), right deletion (–α^3.7^/αα), and the left deletion (–α^4.2^/αα), which account for 60.81% of thalassemia patients (1). In this study, –^SEA^/αα was the most common α-thalassemia genotype account for 52.13%, followed by –α^3.7^/αα (Table 1). This result is consistent with the genotypes of Yulin and Fuzhou areas in China (17, 18), which is also similar to Lao Loum Group in the Lao People's Democratic Republic (19). Additionally, our study showed a high prevalence of –^SEA^/–α^3.7^ mutations account for 16.43%, which was higher than the incidence of the –α^4.2^/αα (0.95%; Table 1). This result is different to the other southern regions in China (20).

In contrast to α-, β-thalassemia carriers are more often caused by point mutations. At present, more than 300 different β-globin gene mutations have been reported around the world (4). In China, more than 40 β-globin gene mutations have been founded, the common mutations are CD41–42, CD17 (A>T), −28 (A>G), and IVS-II-654 (C>T) that account for 86.0% of the cases studied (6). The spectrums of the β-thalassemia gene mutations is diverse in different areas (7). Our data showed that 15 different mutations and 30 genotypes were identified in the 610 β-thalassemia carriers. CD17 (A>T) is the most common β-thalassemia mutation (Table 2), which consist with Guizhou province (21). However, CD41–42 is the most frequent mutation in Guangxi and Hainan in the southern of China (17, 22). While IVS-II-654 were the principal mutations of β-thalassemia in Meizhou city of Guangdong Province (23). In contrast to our study, it is also reported that CD26 (β^E^) is the most frequent mutation in Yunnan region (11). The difference was probably due to the difference of samples and subjects. Additionally, two rare mutations of CD34 and Int were identified in the children according to our knowledge (Table 2), which was the first report of the abnormal hemoglobin in the Chinese population. This results show that there is a great heterogeneity of genotype in β-thalassemia in this area. Yunnan is a multi-ethnic province, thus the heterogeneity were caused by population admixture, marriage patterns, and migrations.

## Conclusion

Our data suggested that the prevalence and gene mutation spectrum of thalassemia display obviously heterogeneity among children in Yunnan Province. The findings extended the mutation spectrum of thalassemia in this region, which provided the valuable information for premarital and pre-pregnancy screening, prenatal diagnostic services, and designing appropriate prevention programs to control the incidence of severe thalassemia for future in this area.

## Data Availability Statement

All datasets generated for this study are included in the article/supplementary material.

## Ethics Statement

The studies involving human participants were reviewed and approved by The Kunming Children's Hospital research ethics committee. Written informed consent to participate in this study was provided by the participants' legal guardian/next of kin.

## Author Contributions

T-LH, T-YZ, and Y-HY designed the study. XT, Q-LL, C-YS, Y-BL, and YL treated patients and helped draft the manuscript. B-HS, C-HY, and NL performed data collection. T-LH and X-WZ analyzed the data and wrote the manuscript.

### Conflict of Interest

The authors declare that the research was conducted in the absence of any commercial or financial relationships that could be construed as a potential conflict of interest.
